# Dermatitis Artefacta, a Form of Factitial Disorder Imposed on Self, Misdiagnosed as Pyoderma Gangrenosum for Eight Years

**DOI:** 10.7759/cureus.9054

**Published:** 2020-07-07

**Authors:** Melissa R Laughter, Aleksandra G Florek, Joshua Wisell, Sabrina Newman

**Affiliations:** 1 Dermatology, University of Colorado School of Medicine, Aurora, USA; 2 Pathology, University of Colorado School of Medicine, Aurora, USA

**Keywords:** pyoderma gangrenosum, factitious ulcer, dermatitis artefacta, facticious disorder, factitial disorder imposed on self

## Abstract

Dermatitis artefacta is a rare psychological disorder in which patients self-inflict cutaneous lesions to satisfy an emotional need. Due to the nature of this disease, patients can present with a wide array of sometimes very severe skin lesions. Here, we describe a case of dermatitis artefacta initially misdiagnosed as pyoderma gangrenosum and treated as such for eight years. The patient reported a long history of cutaneous ulcers on her extremities and trunk, with resultant extensive scarring. Upon presentation, she displayed rapidly progressing necrotizing skin lesions on her bilateral distal lower extremities. Both the skin manifestations and histologic sections were extremely atypical for pyoderma gangrenosum leading to extensive medical records review and subsequent diagnosis of dermatitis artefacta. This case represents the importance of the timely recognition and treatment of dermatitis artifacta to prevent its progression to severe harm and even death.

## Introduction

Dermatitis artefacta (DA) represents a type of factitious disorder within the broad Diagnostic and Statistical Manual of Mental Disorders, Fifth Edition (DSM-5) category of “somatic symptom and related disorders”. Patients inflict cutaneous lesions in order to satisfy a psychological or emotional need of which they are usually not consciously aware [1]. Skin lesions can include blisters, erosions, ulcers, abrasions, edema, erythema, or burn injuries. However, it is crucial to note that the skin lesions can be as varying as the many causes behind them [2]. Here, we present a case of DA initially misdiagnosed as pyoderma gangrenosum (PG) and treated as such for eight years leading to clinical deterioration of the patient.

## Case presentation

**Figure 1:**
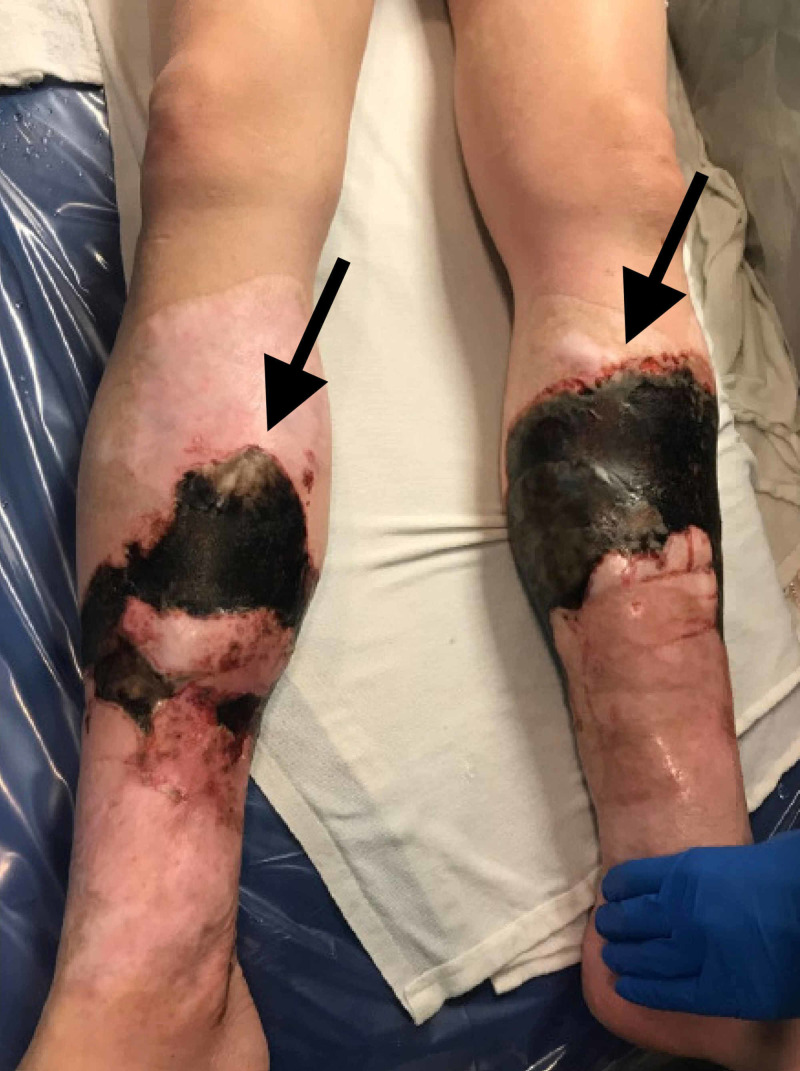
Lower legs showing black necrotic ulcer with well-defined raised erythematous borders.

A 36-year-old female was transferred to the burn unit with rapidly progressing necrotizing skin lesions on her bilateral distal lower extremities. She reported noting small red-brown lesions on her right lower leg that rapidly progressed within 24 hours into black necrotic plaques, circumferential around both of her lower extremities (Figure 1).

Examination revealed discrete hypoesthetic eschar plaques on the bilateral lower distal extremities. Some plaques showed sharp well-demarcated borders and some with branching edges. No retiform purpura, palpable purpura, crepitus, or livedo was noted. Right lower extremity showed a background of atrophic shiny plaques coupled with scars at sites of reported prior PG lesions. She was afebrile with a leukocytosis of 20, C-reactive protein (CRP) of 80 (up from 23 during recent admission), and comprehensive metabolic panel (CMP) within normal limits.

**Figure 2:**
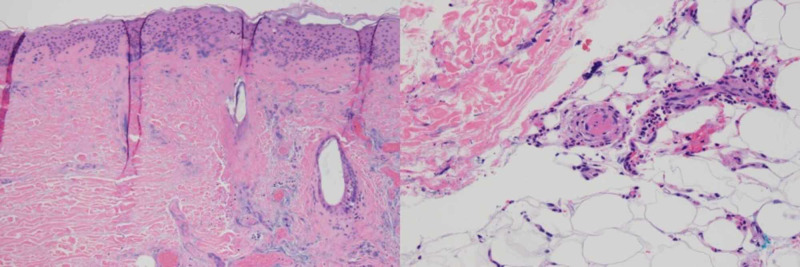
Histologic sections from wedge biopsies taken from the edge of the lesions showing widespread necrosis involving the epidermis and dermis. Necrotic hair follicles are also found on the right side of the image (left) (hematoxylin-eosin, ×10). Small vessel in the superficial subcutis occluded with fibrinous material. Acute inflammation is present in the surrounding adipose tissue (right) (hematoxylin-eosin, ×20).

Histologic sections from wedge biopsies taken from the edge of the lesions showed extensive necrosis involving the epidermis, dermis, and subcutaneous tissue with vascular thrombosis and acute inflammation (Figure 2). Due to this extremely atypical presentation, extensive medical records were reviewed to reveal no confirmed diagnosis of PG, vasculitis, vasculopathy, or associated illnesses. Furthermore, medical records revealed an eight-year history of multiple burns and episodes of ulcerating lesions with little corroborating data regarding diagnosis. Psychiatry was consulted and based on the patient’s exam and history, diagnosed her with DA. The patient received a final diagnosis of chemical burn, full thickness (third degree) of left and right lower leg, and DA.

## Discussion

Although the patient’s self-reported history was significant for PG, the extremely atypical presentation led to consideration of a broader differential diagnosis. PG is a rare, inflammatory dermatosis often associated with systemic disease and is a diagnosis of exclusion. The classic presentation of PG begins as painful erythematous pustules or nodules most often arising on the lower extremities or truck. The lesions then rapidly progress to cutaneous ulcerations with a well-defined, raised inflammatory border and mucopurulent base. Ulcers are exquisitely tender and may expand quickly in one direction and slow in another, leading to a serpiginous pattern [3]. The hypoesthesia and eschar of the plaques experienced by the patient was not typical of the inflammation seen in PG. In addition, PG often shows pathergy, with a propensity to develop within surgical sites and areas of recent skin injury [4]. With such an atypical presentation of PG, it was crucial to keep a broad differential diagnosis that included DA. Unfortunately, the treatment of DA is challenging and often limited by patient compliance. Patients will need close follow-up and care with a multidisciplinary team including dermatology and psychiatry. Treatment of the skin symptoms should involve the appropriate ointments and dressings to improve healing. More comprehensive treatment plans should be tailored to each individual patient to achieve successful outcomes and should involve treatment of the underlying psychiatric disorder [2,5].

## Conclusions

In dermatologic practice, it is important to take note of patients with persistent chronic conditions that do not respond to treatment. Conditions that have atypical presentations should be questioned even further. In this case specifically, the presence of chronic ulcers that were resistant to treatment coupled with a complicated medical history and no confirmed diagnosis should prompt medical record investigation to decrease misdiagnosis of DA and prevent severe self-harm.
